# Activated protein C drives β-arrestin-2- and c-Src-dependent phosphorylation of Cav1 and modulates Cav1 association with PAR1 and GRK5

**DOI:** 10.1016/j.jbc.2026.111190

**Published:** 2026-01-23

**Authors:** Huaping Qin, Lennis B. Orduña-Castillo, Olivia Molinar-Inglis, Monica L. Gonzalez Ramirez, Miguel A. Lopez-Ramirez, Carolyne Bardeleben, JoAnn Trejo

**Affiliations:** 1 Department of Pharmacology, School of Medicine, University of California, San Diego, La Jolla, California, USA; 2 Department of Medicine, School of Medicine, University of California, San Diego, La Jolla, California, USA

**Keywords:** APC, arrestin, biased signaling, caveolin, endothelial, GPCR, GPCR kinase, GRK, protease-activated receptor-1, c-Src, thrombin

## Abstract

G-protein–coupled receptors (GPCRs) display bias toward either G proteins or GPCR kinase (GRK)–mediated β-arrestin (βarr) signaling depending on the agonist-stabilized receptor conformation. The cellular context and subcellular location of GPCRs can also influence biased signaling through mechanisms that are not well understood. The protease-activated receptor-1 (PAR1) exhibits signaling bias in response to thrombin and activated protein C (APC). APC-induced βarr2-biased signaling requires PAR1 compartmentalization in caveolae, a subtype of lipid rafts, whereas thrombin-activated PAR1 G protein signaling does not. Caveolin-1 (Cav1) is the principal structural component of caveolae and regulates protein–protein interactions. The mechanisms by which Cav1 contributes to APC–PAR1-induced βarr2-biased signaling are not known. Here, we report that a substantial population of endogenous PAR1 colocalizes with Cav1 in endothelial cells and is modulated by APC, assessed by single-molecule super-resolution stochastic optical reconstruction microscopy imaging. APC activation of PAR1 also induces Cav1 tyrosine-14 phosphorylation through a βarr2- and c-Src-dependent pathway, which disrupts PAR1–Cav1 coassociation. A smaller population of endogenous GRK5 was also found to colocalize with Cav1 in endothelial cells and was modestly altered by APC activation of PAR1. Moreover, GRK5 was found to interact with Cav1 in intact cells through an N-terminal aromatic-rich Cav1 binding motif. Mutation of this motif disrupts GRK5–Cav1 binding, shifts GRK5 predominantly to the cytoplasm rather than the plasma membrane, and perturbs GRK5-mediated βarr2 recruitment to APC-activated PAR1. Thus, beyond its structural function, Cav1 participates in protein–protein interactions with PAR1 and GRK5, two key effectors that enable APC-induced βarr2 signaling.

Protease-activated receptor-1 (PAR1) exhibits signaling bias in response to activation by the coagulant protease thrombin and the anticoagulant protease activated protein C (APC) (1, 2, 3, 4). In endothelial cells, thrombin activation of PAR1 promotes coupling to heterotrimeric G proteins, leading to barrier disruption and inflammatory responses, such as upregulation of adhesion molecules and cytokine production (5, 6, 7). In contrast, APC activation of PAR1 drives β-arrestin-2 (βarr2)-mediated endothelial cytoprotective responses, including enhanced barrier stabilization, anti-inflammatory, and antiapoptotic prosurvival activities (1, 8, 9). The βarr2 function as the central driver of APC cytoprotection has been verified *in vivo* (10). Thrombin- and APC-biased agonism at PAR1 is enabled through cleavage of the PAR1 N terminus at distinct sites, resulting in the generation of unique N-terminal tails. Thrombin binds directly to PAR1 and cleaves the N terminus at arginine (R)-41, generating an N-terminal tethered ligand that binds intramolecularly to the receptor to activate G protein signaling (11, 12, 13). APC binding to its cofactor endothelial protein C receptor (EPCR), a single transmembrane helix protein, facilitates cleavage of PAR1 at the N-terminal R46 site and drives βarr2-mediated cytoprotective signaling (1, 2, 8, 14).

Caveolae are a subtype of lipid raft plasma membrane microdomains highly abundant in endothelial cells. Previous studies showed that cholesterol-chelating agents disrupt lipid raft localization of PAR1 and EPCR and block APC/PAR1–promoted barrier protection (15, 16). Caveolin-1 (Cav1) is the major structural protein essential for caveolae formation and mediates protein–protein interactions to regulate signaling (17, 18). In addition to lipid raft disruption, we showed that depletion of Cav1 expression blocks APC-activated PAR1 endothelial cytoprotective signaling, whereas thrombin-induced PAR1 signaling remains intact (8, 16). These findings indicate that thrombin- and APC-activated PAR1–biased signaling occurs in distinct compartments, that is, plasma membrane *versus* caveolae microdomains. The mechanisms by which cellular localization of GPCRs in distinct plasma membrane microdomains like caveolae regulates GPCR-biased signaling are poorly understood.

GPCR kinases (GRKs) must localize to the plasma membrane to bind and phosphorylate activated GPCRs and are critical for regulating GPCR-biased signaling (19, 20). GRKs use different mechanisms for membrane localization. GRK2 and GRK3 contain a Pleckstrin homology domain that binds phospholipids and free G protein βγ subunits (21, 22). GRK4 and GRK6 are lipid modified, whereas GRK5 relies primarily on a C-terminal amphipathic helix (23, 24, 25). Besides GPCRs, GRKs are also known to interact with other proteins, including Cav1 (26). All GRKs, including GRK5, harbor an N-terminal caveolin binding motif enriched in aromatic residues. Notably, GRK2 and GRK5 were shown to directly interact *in vitro* with the Cav1 scaffolding domain (CSD) (27). Biased agonists activate different GRKs that promote phosphorylation of GPCRs at distinct sites and elicit unique β-arrestin-mediated responses (28, 29, 30). Unlike classic biased agonists, we recently showed that thrombin- and APC-activated PAR1–biased signaling are regulated by the same GRK, GRK5 (31). This occurs despite the biased agonists requiring distinct localization of PAR1, the plasma membrane for thrombin *versus* caveolae microdomains for APC. Currently, it is not known if Cav1 function is limited to caveolae structural integrity or if Cav1 itself functionally regulates APC-activated PAR1 βarr2–biased signaling.

In this study, we report that APC modulates endogenous PAR1–Cav1 and GRK5–Cav1 colocalization assessed by single-molecule super-resolution stochastic optical reconstruction microscopy (STORM) imaging in cultured human endothelial cells. We also showed that APC promotes Cav1 phosphorylation through a βarr2- and c-Src-mediated pathway and disrupts PAR1–Cav1 coassociation. In addition, we found that the GRK5–Cav1 association is modestly altered by APC, and GRK5 interacts with Cav1 *via* a previously reported N-terminal Cav1 binding motif in intact cells (27). A GRK5 mutant defective in this motif localized predominantly to the cytoplasm rather than the plasma membrane and failed to promote βarr2 recruitment to APC-activated PAR1. Thus, beyond the structural role of caveolin-1, these studies suggest that APC promotes Cav1 phosphorylation and alters Cav1 interaction with PAR1 and GRK5, key effectors that drive APC endothelial cytoprotection.

## Results

### APC-activated PAR1 promotes Cav1 Y14 phosphorylation

**Figure 1:**
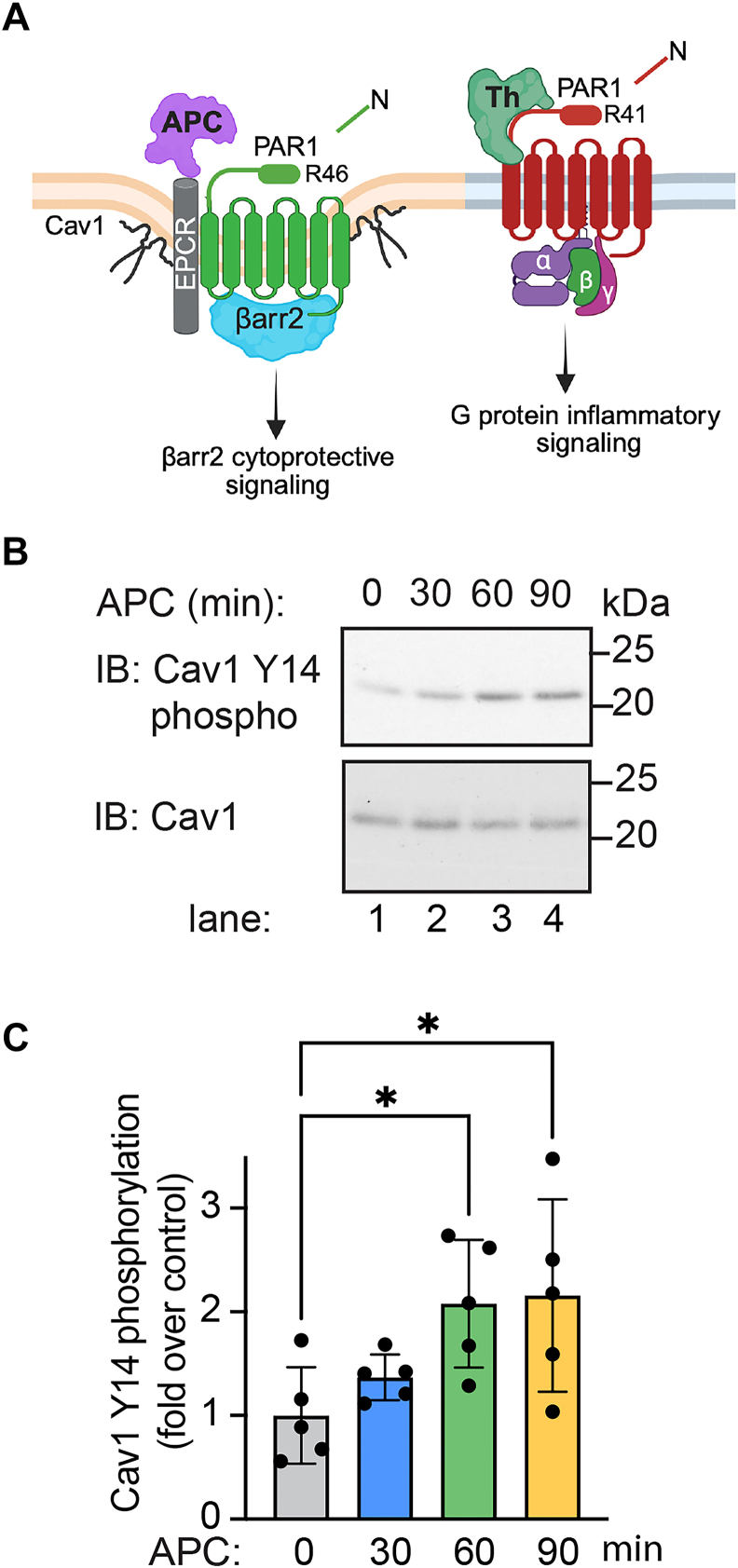
**APC stimulates Cav1 tyrosine (Y)-14 phosphorylation.***A, cartoon* of PAR1-biased signaling. APC bound to the endothelial protein C receptor (EPCR) cofactor activates PAR1 through arginine (R)46 cleavage and promotes βarr2-driven cytoprotective signaling from caveolae microdomains in endothelial cells. Thrombin (Th) cleaves PAR1 at R41 and promotes G protein inflammatory signaling and does not require compartmentalization in caveolae. *B* and *C,* endothelial EA.hy926 cells were treated with APC for various times, and Cav1 Y14 phosphorylation was detected by immunoblotting. Total Cav1 was used as a loading control. Data (mean ± SD) from five independent biological replicates are expressed as the fraction relative to the control (0 min) and analyzed by one-way ANOVA, followed by Dunnett's multiple comparisons test. *C,* ∗*p* = 0.0343 at 60 min; ∗*p* = 0.0229 at 90 min. APC, activated protein C; βarr2, β-arrestin-2; Cav1, caveolin-1; PAR1, protease-activated receptor-1.

Our previous studies indicate that thrombin- and APC-activated PAR1–biased signaling is spatially organized through compartmentalization in distinct plasma membrane microdomains (1, 8, 16). Thrombin-activated PAR1 engages heterotrimeric G proteins at the plasma membrane and promotes inflammatory signaling, whereas APC-activated PAR–induced βarr2-mediated cytoprotective signaling occurs in caveolae (Fig. 1*A*). To understand how Cav1 might contribute to APC-activated PAR1–biased signaling beyond its structural role, we examined Cav1 phosphorylation. Cav1 tyrosine (Y)-14 phosphorylation is a key regulatory site that modulates protein–protein interactions and cellular signaling (17, 32). To determine if APC activation of PAR1 modulates Cav1 Y14 phosphorylation, human umbilical vein endothelial cell–derived EA.hy926 cells were stimulated with APC for various times, and Cav1 Y14 phosphorylation was examined. APC stimulated a significant increase in Cav1 Y14 phosphorylation at 60 min that remained elevated for 90 min (Fig. 1*B*, lanes 1–4, and Fig. 1*C*). The timing of APC-induced Cav1 Y14 phosphorylation is consistent with the kinetics of APC-activated PAR1–induced βarr2-mediated cytoprotective signaling, which displays a slow onset and prolonged response (1, 8). These results suggest that APC may modulate the function of Cav1 *via* phosphorylation to facilitate protein–protein interaction.

### Cav1 phosphorylation induced by APC occurs *via* a c-Src-dependent pathway

**Figure 2:**
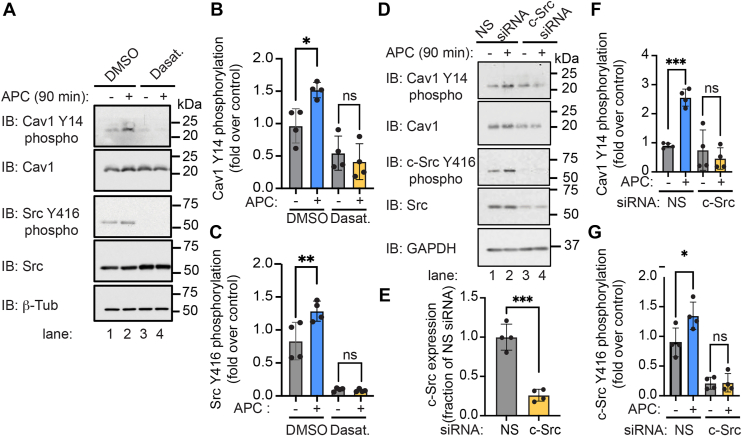
**Cav1 tyrosine (Y)-14 phosphorylation induced by APC–PAR1 is mediated by c-Src.***A*–*C,* endothelial EA.hy926 cells were pretreated with dasatinib or DMSO prior to addition of APC. Cell lysates were then immunoblotted to detect Cav1 Y14 and c-Src Y416 phosphorylation as indicated. *D*–*G,* endothelial cells transfected with nonspecific (NS) or c-Src-specific siRNA were treated with or without APC, lysed, and immunoblotted as indicated. β-tubulin and GAPDH were used as loading controls. The data were quantified (mean ± SD) from four independent biological replicates and expressed as the fraction relative to the untreated control and analyzed by two-way ANOVA followed by Šídák's multiple comparisons test. (*B*) ∗*p* = 0.0136; (*C*) ∗∗*p* = 0.0038; (*F*) ∗∗∗*p* = 0.0003; and (*G*) ∗*p* = 0.0125. Student's unpaired *t* test, (*E*) ∗∗∗*p* = 0.0002. APC, activated protein C; Cav1, caveolin-1; DMSO, dimethyl sulfoxide; PAR1, protease-activated receptor 1.

Src family kinases (SFKs) are known to promote phosphorylation of Cav1 at its Y14 site (32). To determine if SFKs mediate APC-induced Cav1 Y14 phosphorylation, we used dasatinib, a potent SFK pharmacological inhibitor. In control-treated endothelial cells, APC induced a significant increase in Cav1 Y14 phosphorylation that was inhibited in cells preincubated with dasatinib (Fig. 2*A*, *top panels*, lanes 1 and 2 *versus* 3 and 4, and Fig. 2*B*). APC also induced a significant increase in Src Y416 autophosphorylation (Fig. 2*A*, *middle panel*, lanes 1 and 2, and Fig. 2*C*), which results from Src conformational changes and release from an autoinhibited state (33). Dasatinib pretreatment blocked APC-stimulated Src Y416 phosphorylation in endothelial cells (Fig. 2*A*, *middle panel*, lanes 1 and 2 *versus* 3 and 4, and Fig. 2*C*). Next, we determined whether c-Src mediates APC-induced Cav1 Y14 phosphorylation using an siRNA knockdown approach. Expression of c-Src was depleted in cells transfected with the c-Src siRNA compared with nonspecific siRNA–transfected cells (Fig. 2*D*, *lower panel*, lanes 1 and 2 *versus* 3 and 4, and Fig. 2*E*). In nonspecific siRNA–transfected cells, APC induced a significant increase in Cav1 Y14 phosphorylation that was not evident in c-Src-depleted cells (Fig. 2*D*, *top panels*, lanes 1 and 2 *versus* 3 and 4, and Fig. 2*F*). As expected, APC-stimulated c-Src Y416 phosphorylation was abolished in cells lacking c-Src expression (Fig. 2*D*, *middle panel*, lanes 1 and 2 *versus* 3 and 4, and Fig. 2*G*). These data suggest that APC activation of PAR1 promotes c-Src-mediated Cav1 Y14 phosphorylation in endothelial cells.

### βarr2 regulates APC-activated PAR1–induced Cav1 Y14 phosphorylation and c-Src activation

**Figure 3:**
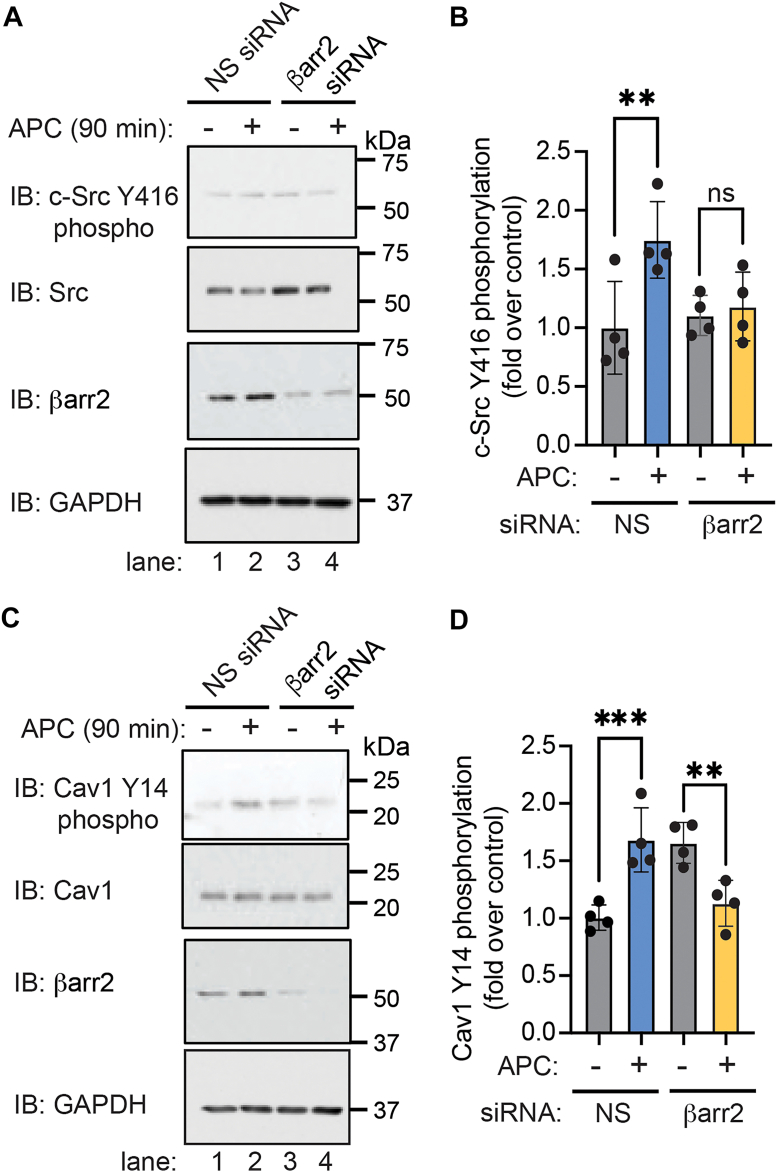
**βarr2 is required for APC-stimulated c-Src tyrosine (Y)-416 phosphorylation and Cav1 Y14 phosphorylation.** Endothelial EA.hy926 cells were transfected with nonspecific (NS) or βarr2-specific siRNA, treated with APC, and c-Src Y416 phosphorylation (*A* and *B*) and Cav1 Y14 phosphorylation (*C* and *D*) were detected by immunoblotting as indicated. GAPDH was used as a loading control. The data were quantified (mean ± SD) from four independent biological replicates and expressed as the fraction relative to the untreated control and analyzed by two-way ANOVA followed by Šídák's multiple comparisons test. *B,* NS siRNA with and without APC, ∗∗*p* = 0.0097; ns = not significant. *D,* NS siRNA with and without APC ∗∗∗*p* = 0.0009; βarr2 siRNA with and without APC, ∗∗*p* = 0.0061. APC, activated protein C; βarr2, β-arrestin-2; Cav1, caveolin-1.

βarr2 is the major transducer of APC-activated PAR1 cytoprotective signaling (1, 8), and β-arrestins have been reported to regulate c-Src activity (34, 35). These studies suggest that βarr2 may be required for c-Src-mediated Cav1 Y14 phosphorylation induced by APC activation of PAR1. In nonspecific siRNA–transfected endothelial cells, APC induced a marked increase in c-Src Y416 phosphorylation that was markedly inhibited in βarr2 siRNA–transfected cells (Fig. 3*A*, lanes 1 and 2 *versus* 3 and 4, and Fig. 3*B*). Thus, βarr2 is required for APC-activated PAR1–induced c-Src activation in endothelial cells. Next, we examined the role of βarr2 in APC-induced Cav1 Y14 phosphorylation. APC induced a significant increase in Cav1 Y14 phosphorylation in nonspecific siRNA–transfected cells that was significantly decreased in βarr2-deficient cells (Fig. 3*C*, lanes 1 and 2 *versus* 3 and 4, and Fig. 3*D*). Unexpectedly, however, basal Cav1 Y14 phosphorylation was increased in cells lacking βarr2 (Fig. 3*C*, lanes 1 *versus* 3, and Fig. 3*D*). The change in basal Cav1 Y14 phosphorylation may result from disruption of important regulatory protein–protein interactions and/or be related to the modest increase in Src expression observed in βarr2-depleted cells (Fig. 3*A*, lanes 3 and 4 *versus* 1 and 2). Nonetheless, these findings suggest that APC-activated PAR1 promotes Cav1 Y14 phosphorylation through a βarr2- and c-Src-dependent pathway.

### APC regulates PAR1–Cav1 colocalization assessed by STORM imaging, and coassociated PAR1–Cav1 complexes are regulated by c-Src

**Figure 4:**
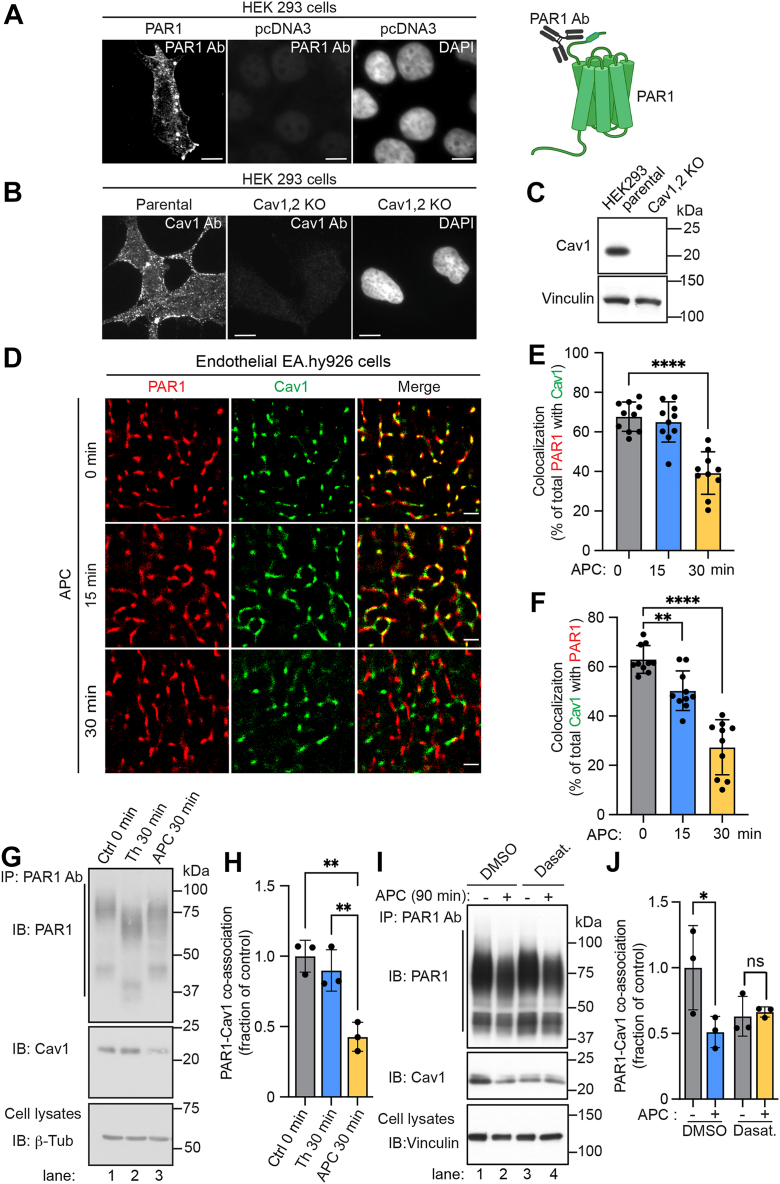
**APC modulates endogenous PAR1–Cav1 colocalization and coassociation *via* a c-Src-dependent pathway.***A,* HEK293 cells transfected with human PAR1 or pcDNA3 plasmids were immunostained with the anti-PAR1 WEDE antibody (Ab), which recognizes the N terminus of PAR1 (*cartoon*). PAR1 expression was detected by immunofluorescence confocal microscopy. The scale bar represents 100 μm. *B,* HEK293 parental cells and Cav1,2 KO cells were immunostained for Cav1 and imaged by confocal microscopy. The scale bar represents 100 μm. *C,* HEK293 parental cells and Cav1,2 KO cell lysates were immunoblotted for Cav1. Vinculin was used as a loading control. *D,* endothelial EA.hy926 cells were incubated with or without APC for various times, processed, immunostained for PAR1 (*red*) and Cav1 (*green*), and imaged by STORM. The scale bar represents 1 μm. *E* and *F,* PAR1–Cav1 colocalization was quantified (mean ± SD) from three independent biological experimental replicates using 10 cells per condition per experiment and expressed as a percent of total PAR1 with Cav1 and total Cav1 with PAR1. Data were analyzed by one-way ANOVA, followed by Tukey's multiple comparisons test. *E,* ∗∗∗∗*p* < 0.0001and *F*, ∗∗*p* = 0.0073 and ∗∗∗∗*p* < 0.0001. *G,* endothelial EA.hy926 cells were treated with or without APC or thrombin (Th) or (*I*) pretreated with dasatinib or DMSO, followed by APC as indicated, then were immunoprecipitated (IP) with anti-PAR1 antibodies (Abs) and endogenous PAR1–Cav1 coassociation was detected by immunoblotting. β-tubulin or vinculin was used as a loading control. *H* and *J,* PAR1–Cav1 coassociation was quantified (mean ± SD) from three independent biological replicates and expressed as a fraction over the untreated control (0 min) and analyzed by one-way ANOVA, followed by Tukey's (*H*) multiple comparisons test, ∗∗*p* = 0.0011 (Ctl *versus* APC); ∗∗*p* = 0.0030 (APC *versus* Th) or two-way ANOVA, followed by Šídák's (*J*) multiple comparisons test, ∗*p* = 0.0254; ns, not significant. APC, activated protein C; Cav1, caveolin-1; DMSO, dimethyl sulfoxide; HEK293, human embryonic kidney 293 cell line; PAR1, protease-activated receptor-1; STORM, stochastic optical reconstruction microscopy.

To determine if APC modulates Cav1 protein–protein interactions, we examined endogenous PAR1 and Cav1 colocalization using STORM imaging combined with total internal reflection fluorescence (TIRF) microscopy that enables illumination of structures at the cell membrane. STORM permits high nanometer scale (20–30 nm) resolution and enables visualization of endogenous PAR1 and Cav1 single molecules in caveolae at or near the plasma membrane. The specificity of the anti-PAR1 antibody was first validated using human embryonic kidney 293 (HEK293) cells transiently transfected with human PAR1 or pcDNA3 vector. The anti-Cav1 antibody was validated in HEK293 CRISPR–Cas9 Cav1,2 KO cells. In HEK293 cells transiently expressing PAR1, the anti-PAR1 WEDE antibody raised against the receptor N-terminal domain detected PAR1 at the cell surface and in endocytic vesicles, whereas no signal was observed in pcDNA3-transfected control cells (Fig. 4*A*). HEK293 parental cells immunostained with anti-Cav1 antibodies revealed discrete caveolae-like puncta enriched in Cav1 predominantly at the cell periphery that were absent in the Cav1,2 KO cells (Fig. 4*B*). The specificity of the anti-Cav1 antibody was further confirmed by immunoblotting lysates prepared from HEK293 parental and Cav1,2 KO cells (Fig. 4*C*). These analyses indicate that PAR1 and Cav1 antibodies are specific.

Using the validated antibodies, the effect of APC on endogenous PAR1–Cav1 colocalization in endothelial EA.hy926 cells was then examined by STORM imaging. In unstimulated cells, a substantial population of colocalized PAR1–Cav1 molecules was detected; ∼70% of PAR1 colocalized with Cav1 and ∼60% of Cav1 colocalized with PAR1 (Fig. 4, *D*–*F*). The extent of PAR1–Cav1 colocalization was not significantly altered after a 15 min incubation with APC, which remained comparable to untreated controls (Fig. 4, *D*–*F*). A significant decrease in PAR1–Cav1 colocalization was observed following 30 min of APC stimulation as reflected by a reduction from ∼70% to ∼40% for PAR1–Cav1 colocalization and ∼60% to ∼30% for Cav1–PAR1 colocalization (Fig. 4, *D*–*F*), indicating that a considerable decrease in colocalization of the PAR1–Cav1 population was induced by APC.

To substantiate the impact of APC on PAR1–Cav1 protein–protein interaction in endothelial cells, coimmunoprecipitation (co-IP) was used to determine coassociation. Endothelial cells were either left untreated or treated with thrombin or APC, PAR1 was immunoprecipitated with anti-PAR1 antibodies, and coassociated Cav1 was detected. In untreated control cells, PAR1 coassociated basally with Cav1 (Fig. 4*G*, lane 1, and Fig. 4*H*), consistent with the STORM imaging results shown above. After thrombin incubation, activated PAR1 migrated as a lower molecular weight species because of cleavage of the N terminus and retained binding to Cav1 comparable to that observed in untreated control cells (Fig. 4*G*, lanes 1 and 2, and Fig. 4*H*). In contrast, APC-activated PAR1 exhibited a more modest size shift of the cleaved receptor and showed a significant ∼50% decrease in PAR1–Cav1 coassociation, suggesting significant disruption of the PAR1–Cav1 complex (Fig. 4*G* lanes 2 *versus* 3, and Fig. 4*H*). To determine if APC-mediated disruption of PAR1–Cav1 interaction is dependent on c-Src activation, endothelial cells were pretreated with the Src inhibitor, dasatinib. In control cells, PAR1–Cav1 coassociation was significantly disrupted following incubation with APC (Fig. 4*I*, lanes 1 and 2, and Fig. 4*J*), whereas in dasatinib-pretreated cells, APC failed to alter PAR1–Cav1 coassociation (Fig. 4*I*, lanes 3 and 4 *versus* 1 and 2, and Fig. 4*J*), although basal PAR1–Cav1 coassociation was diminished compared with vehicle-treated control cells. Together, these results suggest that APC-activated PAR1 induces Cav1 Y14 phosphorylation *via* a c-Src-dependent pathway and disrupts PAR1–Cav1 coassociation.

### APC regulates endogenous GRK5–Cav1 colocalization but not GRK5–Cav1 protein complexes

**Figure 5:**
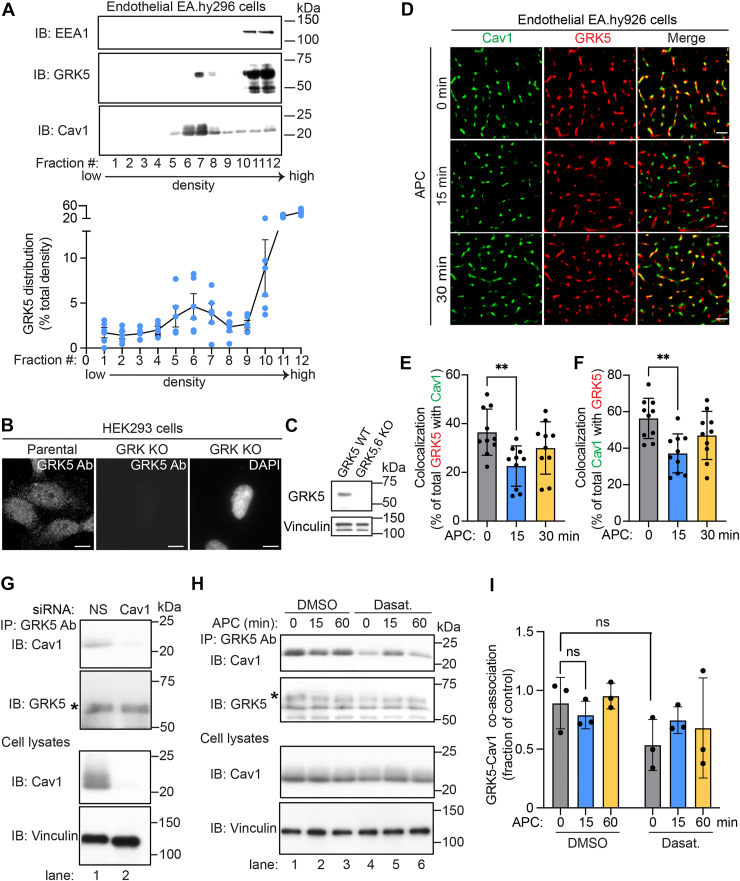
**Endogenous GRK5 localizes to plasma membrane caveolae microdomains in endothelial cells.***A,* endothelial EA.hy926 cell lysates were subjected to sucrose density gradient fractionation, and collected fractions were immunoblotted for Cav1, GRK5, and early endosomal antigen-1 (EEA1) as indicated. The data (mean ± SD) from six independent biological replicates are expressed as the percentage of GRK5 in each fraction compared with the total combined GRK5 signal intensity across all fractions. *B,* specificity of the GRK5 antibody was validated by immunofluorescence using HEK293 parental and CRISPR–Cas9 GRK KO cells. The scale bar represents 100 μm. *C,* immunoblot of lysates from HEK293 parental and GRK5,6 KO cells confirming GRK5 antibody specificity. Vinculin was used as a loading control. *D,* EA.hy926 cells were treated with APC for various times, processed, immunostained for Cav1 (*green*) and GRK5 (*red*), and imaged by STORM. The scale bar represents 1 μm. *E* and *F,* GRK5–Cav1 and Cav1–GRK5 colocalization was quantified (mean ± SD) from three independent biological experimental replicates using 10 cells per condition per experiment and expressed as a percent of total GRK5 colocalized with Cav1 and total Cav1 colocalized with GRK5. Data were analyzed by one-way ANOVA, followed by Tukey's multiple comparisons test. *E,* ∗∗*p* = 0.0084 (GRK5–Cav1) and *F,* ∗∗*p* = 0.0029 (Cav1–GRK5). *G,* endothelial EA.hy926 cells transfected with nonspecific (NS) or Cav1-specific siRNA were processed and immunoblotted as indicated. An *asterisk* indicates GRK5. *H,* endothelial cells were pretreated with dasatinib or DMSO, followed by incubation with APC as indicated, then immunoprecipitated (IP) with anti-GRK5 antibodies (Abs) and endogenous GRK5–Cav1 coassociation detected by immunoblotting. An *asterisk* indicates GRK5. Vinculin was used as a loading control. *I,* GRK5–Cav1 coassociation was quantified (mean ± SD) from four independent biological replicates and expressed as a fraction over untreated control (0 min) and analyzed by two-way ANOVA, followed by Tukey's multiple comparisons test, ns, not significant. APC, activated protein C; Cav1, caveolin-1; DMSO, dimethyl sulfoxide; GRK5, GPCR kinase 5; HEK293, human embryonic kidney 293 cell line; STORM, stochastic optical reconstruction microscopy.

GRK5 was recently shown to regulate APC-activated PAR1–induced recruitment of βarr2 (31) and harbors an aromatic-rich binding motif that facilitates interaction with Cav1 *in vitro* (27). However, it is not known if endogenous GRK5 resides in caveolae in intact cells, and this was examined. Sucrose density gradient fractionation was used to isolate detergent-resistant membranes enriched in Cav1 that separate to lower density fractions. GRK5 was detected in both the Cav1-enriched fractions and heavier fractions containing the early endosome antigen-1 peripheral membrane protein (Fig. 5*A*). Thus, like PAR1 (1, 8), GRK5 is localized both within and outside Cav1-enriched membrane microdomains in endothelial cells.

STORM imaging was next used to directly visualize endogenous GRK5 localization in caveolae. The anti-GRK5 antibody was validated in HEK293 CRISPR–Cas9 GRK KO cells lacking GRK2,3 and 5,6 isoforms by immunofluorescence confocal microscopy. The antibody detected GRK5 in HEK293 parental cells but not in GRK KO cells (Fig. 5*B*). Consistent with confocal imaging, the antibody detected GRK5 expression in HEK293 parental cells but not in the GRK5,6 isoform–specific KO cell lysates (Fig. 5*C*). These results suggest that the antibody specifically and reliably detects the GRK5 protein (36). In untreated control cells, endogenous Cav1 and GRK5 showed substantial colocalization, with ∼38% of GRK5 colocalized with Cav1 and ∼58% of Cav1 colocalized with GRK5 in endothelial cells detected by STORM imaging (Fig. 5, *D*–*F*). After stimulation with APC for 15 min, GRK5–Cav1 coassociation was significantly decreased, as indicated by a reduction from 38% to 22% and from 58% to 38% for Cav1–GRK5 colocalization (Fig. 5, *D*–*F*), resulting in a modest decrease in GRK5 and Cav1 colocalization. However, after 30 min of APC stimulation, the extent of GRK5–Cav1 and Cav1–GRK5 colocalization returned to levels comparable with that observed basally in unstimulated cells (Fig. 5, *D*–*F*). We next determined if GRK5–Cav1 protein complexes were modulated by APC using a co-IP assay. Cav1 was detected in GRK5 IPs from nonspecific siRNA-transfected cells and not in cells depleted of Cav1 (Fig. 5*G*, lanes 1 and 2). Using this co-IP assay, we then examine if GRK5–Cav1 protein complexes were altered following APC incubation and regulated by c-Src. In control pretreated cells, GRK5–Cav1 coassociation was detected and appeared to modestly decrease following 15 min of APC stimulation (Fig. 5*H*, lanes 1 *versus* 2); however, the effect was not statistically significant (Fig. 5*I*). In addition, c-Src inhibition with dasatinib appeared to reduce GRK5–Cav1 basal interaction and to increase GRK5–Cav1 coassociation at 15 min (Fig. 5*H*, lanes 1–3 *versus* 4–6, and Fig. 5*I*), but the apparent effects were not statistically significant. The discrepancy between the effect of APC on GRK5–Cav1 colocalization and coassociation may result from the modest effect of APC on GRK5–Cav1 protein–protein interaction and reduced sensitivity of the co-IP assay. Nonetheless, these findings indicate that GRK5 resides in caveolae, and GRK5–Cav1 colocalization appears to be modestly regulated by APC, an event that occurs prior to APC-induced disruption of PAR1–Cav1 complex.

### A GRK5 Cav1 binding motif mutant displays altered localization to the plasma membrane

**Figure 6:**
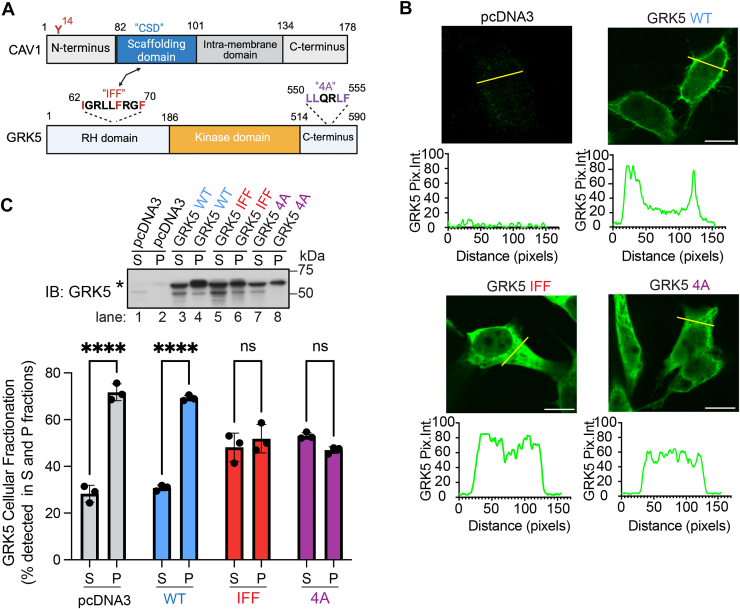
**The GRK5 Cav1 binding IFF motif is important for membrane localization.***A,* schematic of Cav1 (amino acids 1–178) and GRK5 (amino acids 1–590) protein domains. Cav1 contains an N-terminal tyrosine (Y)-14 residue, Cav1 scaffolding domain (CSD), intramembrane domain, and C terminus. GRK5 contains a consensus Cav1 binding motif (residues 62–70) within the RH domain and a kinase domain and C terminus. The putative Cav1 binding motif interaction with the Cav1 scaffolding domain is indicated by the *double-headed arrow*. The GRK5 IFF residues mutated to alanine are highlighted in *red*. *B,* HeLa cells transiently expressing GRK5 WT, IFF, or 4A mutants or pcDNA3 were immunostained and imaged by confocal microscopy. The scale bar represents 100 μm. GRK5 subcellular localization was quantified by line scan analysis of pixel intensity using ImageJ software. *C,* GRK5 WT, IFF, 4A mutants, or pcDNA3 were transfected into HEK293 cells and subjected to cellular fractionation, resulting in a soluble (S)/cytosolic fraction and a particulate (P)/membrane fraction. GRK5 (∗, *asterisk*) was detected by immunoblotting and quantified. The data (mean ± SD) expressed the percent of GRK5 detected in soluble and particulate fractions was determined from three independent biological replicates and analyzed by two-way ANOVA, followed by Šídák's multiple comparisons test, pcDNA3, ∗∗∗∗*p* < 0.0001 (S *versus* P); GRK5 WT, ∗∗∗∗*p* < 0.0001 (S *versus* P). Cav1, caveolin-1; GRK5, GPCR kinase 5; HEK293, human embryonic kidney 293 cell line.

Cav1 and GRK5 have been reported to possess protein domain determinants that facilitate protein–protein interaction (27). Cav1 contains an N-terminal caveolin scaffolding domain that mediates protein–protein interactions that can be modulated by Y14 phosphorylation (37), an interdomain region that embeds in the plasma membrane, and a C terminus that is extensively palmitoylated (Fig. 6*A*) (38). GRK5 contains an N-terminal RH domain, a kinase domain, and a C terminus with an amphipathic helix important for membrane anchoring (Fig. 6*A*) (24, 39). GRK5 has been shown to directly bind to Cav1 *in vitro* through an N-terminal region containing a Cav1-binding aromatic-rich motif I^62^GRLLFRQF^70^ that interacts with the Cav1 CSD (27) (Fig. 6*A*). To determine whether the aromatic-rich Cav1 binding motif is important for GRK5–Cav1 interaction in intact cells, a GRK5 mutant containing isoleucine (I)62 and phenylalanine (F)67 and F70 to alanine (A) conversions was generated and termed the GRK5 “IFF” mutant. The expression and localization of the GRK5 IFF mutant were first examined by immunofluorescence confocal microscopy in HeLa cells. GRK5 WT is known to localize to the plasma membrane *via* a C-terminal amphipathic helix and was found predominantly at the plasma membrane in HeLa cells (Fig. 6B). Unexpectedly, the GRK5 IFF mutant, which retains its amphipathic helix, localized primarily in the cytoplasm, similar to a previously reported GRK5 4A amphipathic helix mutant harboring leucine (L) to alanine mutations at positions L550A, L551A, L554A, and F555A (24) (Fig. 6*B*). To verify GRK5 IFF mislocalization from the plasma membrane, cell lysates were fractionated by high-speed ultracentrifugation into soluble/cytosolic proteins and particulate/membrane protein fractions. A significantly greater amount of GRK5 WT was detected in the particulate membrane fraction compared with the soluble fraction, like endogenous GRK5 detected in these HEK293 cells (Fig. 6*C*, lanes 1 and 2 *versus* 3 and 4). However, both the GRK5 IFF and 4A mutant showed a greater distribution to the soluble cytosolic fraction compared with GRK5 WT (Fig. 6*C*, lanes 5–8 *versus* 3 and 4). These results suggest that the GRK5 IFF mutant is mislocalized and resides predominantly in the cytoplasm, like the previously reported GRK5 4A amphipathic mutant (24).

### A Cav1 binding motif is required for GRK5–Cav1 interaction in cells

**Figure 7:**
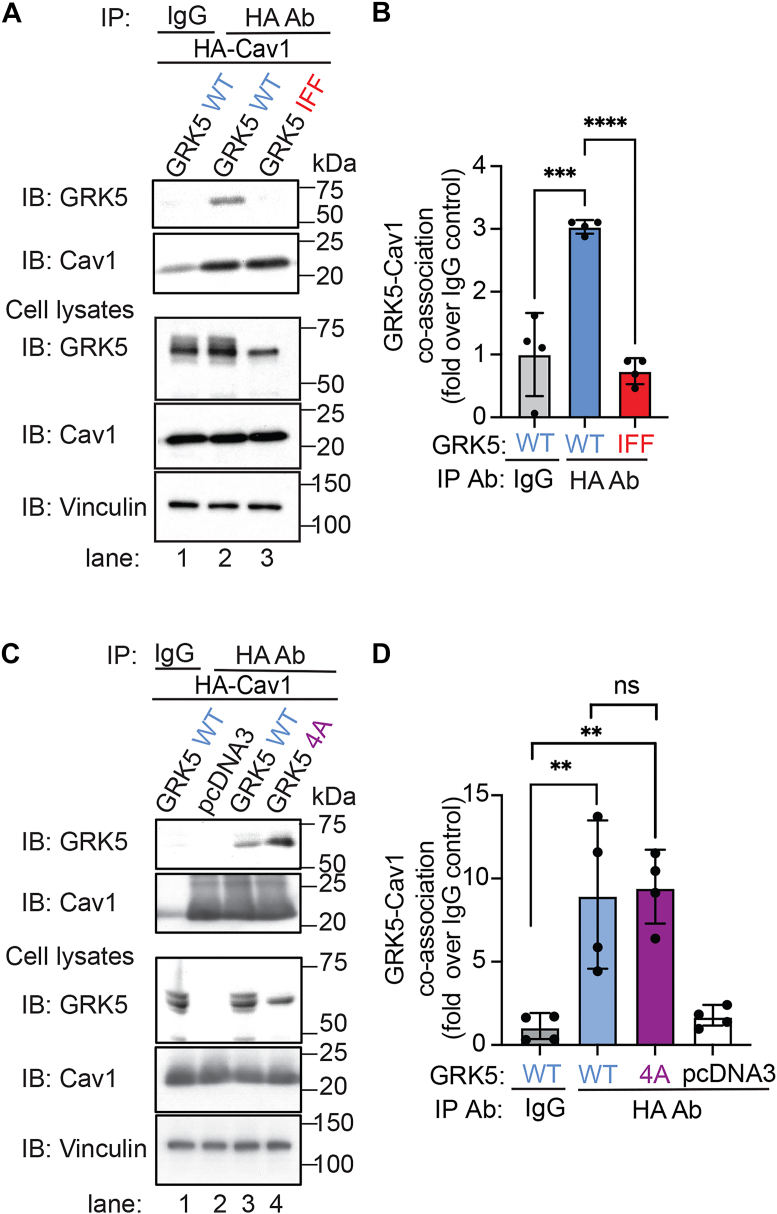
**The GRK5 IFF motif mediates interaction with Cav1 in intact cells.***A* and *C,* HEK293 cells transiently expressing HA-Cav1 together with either GRK5 WT, mutants, or pcDNA3 were immunoprecipitated and immunoblotted as indicated. Cell lysates were immunoblotted for total HA-Cav1, GRK5, and vinculin expression. *B* and *D,* the data (mean ± SD) from four independent biological replicates are expressed as fold over IgG control and were analyzed by one-way ANOVA, followed by Tukey's multiple comparisons test. *B,* ∗∗∗*p* = 0.0002 (IgG *versus* HA antibody [Ab] WT); ∗∗∗∗*p* < 0.0001 (HA Ab WT *versus* IFF). *D,* ∗∗*p* = 0.0073 (IgG *versus* HA Ab WT), ∗∗*p* = 0.0036 (IgG *versus* HA Ab 4A), ns = not significant. Cav1, caveolin-1; GRK5, GPCR kinase 5; HEK293, human embryonic kidney 293 cell line.

Next, we evaluated whether GRK5 WT and IFF mutant interact with Cav1 in HEK293 cells using co-IP. WT GRK5 robustly coassociated with HA-tagged Cav1 in HA *versus* IgG control immunoprecipitates (Fig. 7*A*, lanes 1 and 2, and Fig. 7*B*). In contrast to GRK5 WT, the GRK5 IFF mutant showed a significant reduction in Cav1 interaction in immunoprecipitates (Fig. 7*A*, lanes 2 and 3, and Fig. 7*B*), suggesting that the conserved Cav1 binding IFF motif is critical for GRK5–Cav1 interaction in intact cells. To determine whether the loss of GRK5 membrane anchoring observed with the GRK5 IFF mutant contributed to the loss of Cav1 binding, we examined Cav1 interaction with the GRK5 4A amphipathic helix mutant. In contrast to the GRK5 IFF mutant, the GRK5 4A mutant retained its capacity to interact with Cav1 comparable to GRK5 WT, despite being localized primarily in the cytoplasm (Fig. 7*C*, lanes 3 and 4 *versus* 1 and 2, and Fig. 7*D*). Therefore, the loss of GRK5 membrane anchoring is not sufficient to disrupt GRK5–Cav1 interaction in these cells.

### GRK5 WT, but neither the IFF nor the 4A mutant, supports APC-induced βarr2 recruitment to activated PAR1

**Figure 8:**
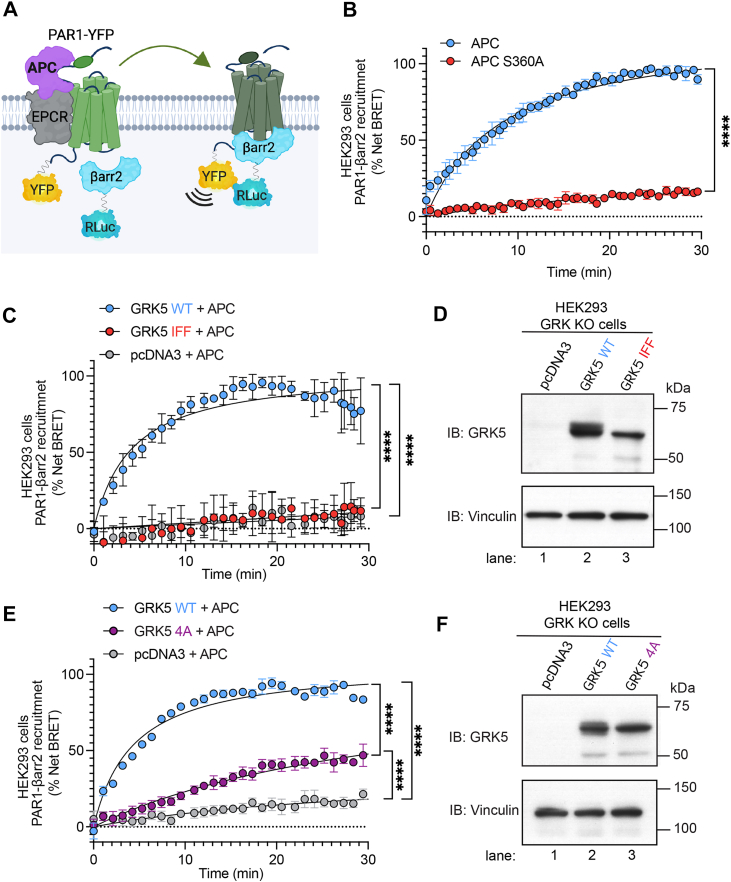
**GRK5 WT, but neither the GRK5 IFF nor the 4A mutant supports APC-activated PAR1–induced βarr2 recruitment.***A, cartoon*, APC bound to EPCR activates PAR1–YFP, resulting in the recruitment of Rluc–βarr2 in the BRET assay. *B,* HEK293 cells transiently expressing PAR1-YFP, EPCR-Halo, and Rluc–βarr2 were treated with 20 nM APC or APC S360A proteolytically inactive mutant, and net BRET was determined. Data (mean ± SD) from three independent biological replicates were analyzed by Student's unpaired *t* test, ∗∗∗∗*p* < 0.0001. HEK293 CRISPR–Cas9 GRK KO cells transiently expressing PAR1-YFP, EPCR-Halo, Rluc–βarr2, and either GRK5 WT, GRK5 IFF mutant, or pcDNA3 (*C* and *D*) or GRK5 WT, GRK5 4A mutant, or pcDNA3 (*E* and *F*) were stimulated with 20 nM APC, and net BRET was determined. Cell lysates were immunoblotted for GRK5 and vinculin expression. Data (mean ± SD) from three or four independent biological replicates were analyzed by one-way ANOVA, followed by Tukey's multiple comparisons test. *C,* ∗∗∗∗*p* < 0.0001 (WT *versus* IFF) and ∗∗∗∗*p* = 0.0001 (WT *versus* pcDNA3). *E,* ∗∗∗∗*p* < 0.0001 (WT *versus* 4A), ∗∗∗∗*p* < 0.0001 (WT *versus* pcDNA3), and ∗∗∗∗*p* < 0.0001 (4A *versus* pcDNA3). APC, activated protein C; βarr2, β-arrestin-2; BRET, bioluminescence resonance energy transfer; EPCR, endothelial protein C receptor; HEK293, human embryonic kidney 293 cell line; PAR1, protease-activated receptor-1; Rluc, Renilla luciferase; YFP, yellow fluorescent protein.

APC-activated PAR1 requires GRK5 for recruitment of βarr2 (31), and βarr2 drives endothelial cytoprotection (1, 8). However, it is not known whether GRK5–Cav1 interaction is important for APC-activated PAR1–induced βarr2 recruitment, and this was examined using bioluminescence resonance energy transfer (BRET) assays. HEK293 cells coexpressing PAR1 fused to yellow fluorescent protein (YFP), the APC coreceptor EPCR, and βarr2-fused *Renilla* luciferase (Rluc) were stimulated with either APC WT or the S360A inactive variant (40), and βarr2 recruitment was monitored by measuring changes in BRET (Fig. 8*A*). APC induced a hyperbolic increase in βarr2 recruitment measured as an increase in net BRET in HEK293 cells (Fig. 8*A*), whereas the APC proteolytically inactive S360A variant failed to promote βarr2 recruitment (Fig. 8*B*). Thus, the HEK293 cell model system recapitulates the requirement for APC proteolytic activation of PAR1 to induce βarr2 recruitment as we previously reported in endothelial cells (16).

**Figure 9:**
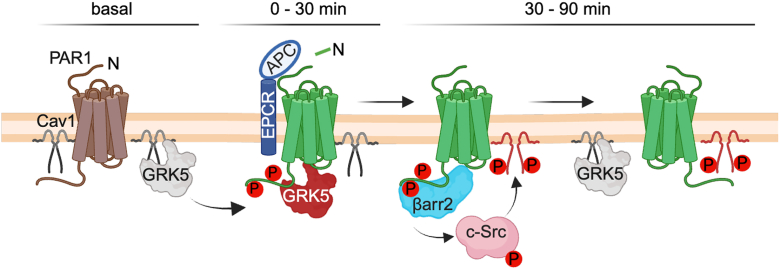
**Model of PAR1–GRK5–βarr2–c-Src regulation by Cav1.** A substantial population of PAR1–Cav1 and GRK5–Cav1 complexes coexist at the plasma membrane under basal conditions. APC bound to EPCR cleaves and activates PAR1, resulting in GRK5-dependent phosphorylation of the receptor C-terminal tail within 30 min. This may occur during a time when GRK5 dissociates from Cav1. Next, APC-activated and phosphorylated PAR1 recruits βarr2, which promotes activation of c-Src Y416 phosphorylation and c-Src-dependent Cav1 Y14 phosphorylation, a process that occurs after 30 min and is sustained through 90 min. At 30 min, GRK5–Cav1 may reassociate, whereas activated PAR1 and Y14 phosphorylated Cav1 remain dissociated through the 90 min interval. APC, activated protein C; βarr2, βarr2; Cav1, caveolin-1; EPCR, endothelial protein C receptor; GRK5, GPCR kinase 5; PAR1, protease-activated receptor-1.

To examine if the GRK5 IFF mutant promoted APC-induced βarr2 recruitment similar to WT GRK5, we used HEK293 CRISPR–Cas9 GRK KO cells expressing PAR1-YFP, the APC coreceptor EPCR, and βarr2–Rluc together with GRK5 WT, IFF, or 4A mutant and assessed the effect on APC-induced βarr2 recruitment using BRET. In cells coexpressing GRK5 WT, APC stimulated an increase in βarr2 recruitment to activated PAR1 (Fig. 8, *C* and *D*, lane 2), whereas APC failed to stimulate recruitment of βarr2 in pcDNA3 vector–transfected cells (Fig. 8, *C* and *D*, lanes 2 *versus* 1). These findings indicate that GRK5 expression is critical for APC-induced βarr2 recruitment. In contrast to WT GRK5, expression of the GRK5 IFF mutant failed to restore APC-induced βarr2 recruitment to activated PAR1 (Fig. 8, *C* and *D*, lanes 2 *versus* 3). Similarly, in cells expressing the GRK5 4A mutant, APC stimulated a modest quasi-linear increase in βarr2 recruitment (Fig. 8, *E* and *F*, lanes 1–3), consistent with a critical function for GRK5 membrane anchoring in facilitating APC-induced βarr2 recruitment as recently reported (31). These results indicate that although GRK5–Cav1 interaction may contribute to βarr2 recruitment, proper GRK5 membrane localization is likely most critical for APC-activated PAR1–induced βarr2 recruitment. Collectively, these findings indicate that endogenous PAR1–Cav1 and GRK5–Cav1 molecules basally colocalize at the plasma membrane. Upon APC stimulation, GRK5 dissociates from Cav1 and mediates PAR1 phosphorylation within 30 min. Activated and phosphorylated PAR1 then recruits βarr2 (31), which promotes c-Src activation, resulting in Cav1 Y14 phosphorylation and disruption of PAR1–Cav1 colocalization and protein complexes that occur over the next 30 to 90 min time frame (Fig. 9).

## Discussion

Expression of Cav1 is essential for APC-activated PAR1–induced endothelial cytoprotection (8, 16). However, the mechanisms underlying Cav1 requirement for APC-activated PAR1 cytoprotective signaling are not known. Here, we report that a substantial population of endogenous PAR1 and a smaller population of GRK5 colocalize with Cav1 molecules as revealed by STORM imaging, indicating that these proteins reside in caveolae basally. In addition, APC activation of PAR1 decreased endogenous PAR1–Cav1 colocalization and disrupted PAR1–Cav1 complexes *via* a mechanism that requires βarr2- and c-Src-dependent Cav1 Y14 phosphorylation in endothelial cells. We also found that endogenous GRK5–Cav1 molecules are modestly modulated by APC using STORM imaging but not in biochemical co-IP assays used to assess coassociation. Finally, our results show that a GRK5 mutant defective in Cav1 binding and plasma membrane anchoring fails to support APC-activated PAR1–induced βarr2 recruitment, unlike WT GRK5. Thus, beyond its structural role, Cav1 phosphorylation and modulation of protein–protein interactions with PAR1 and GRK5 are likely critical for APC-activated PAR1–induced cytoprotective signaling.

Caveolae are a subtype of lipid rafts enriched in cholesterol, highly abundant in endothelial cells, and required for APC-activated PAR1 cytoprotective signaling. The formation of caveolae in endothelial cells requires Cav1, the principal structural component, and cholesterol (17). Studies showed that depletion of cholesterol using methyl-β-cyclodextrin, which perturbs both lipid rafts and caveolae, disrupts APC-activated PAR1 signaling in endothelial cells (15, 16). To assess the role of caveolae directly, Cav1-specific shRNA and siRNA targeting strategies were taken and shown to abolish Cav1 expression and block APC/PAR1-mediated endothelial barrier protection and antiapoptotic responses (8, 16). In addition, PAR1 was shown to localize in Cav1-enriched membranes in sucrose density gradient fractionation studies (8, 16). However, direct visual evidence that endogenous PAR1 localizes to caveolae in intact endothelial cells has not been previously reported. Caveolae are small plasma membrane invaginations (50–100 nm) that require super-resolution imaging for visualization (17). In this study, we utilized STORM TIRF imaging to directly visualize caveolae and found that 70% of endogenous PAR1 molecules colocalize with Cav1 at the plasma membrane. These studies provide strong evidence that under basal conditions, a substantial population of endogenous PAR1 molecules resides in caveolae in endothelial cells.

Cav1 Y14 phosphorylation induces conformational changes that spatially separate Cav1 molecules and may alter the conformation or accessibility of the Cav1 CSD (32, 41). The Cav1 CSD mediates protein–protein interactions through recognition of caveolin binding motifs originally identified as aromatic-rich amino acid sequences that are uniquely spaced (ØXØXXXXØ, ØXXXXØXXØ, or ØXØXXXXØXXØ, where Ø is Trp, Phe, or Tyr) (42). Several CSD protein–protein interactions are regulated by Cav1 Y14 phosphorylation, including Cav1 CSD–focal adhesion interactions (43) and Cav1 CSD–endothelial nitric oxide synthase coassociation (44, 45). Here, we report that APC promotes Cav1 Y14 phosphorylation through a βarr2- and c-Src-dependent pathway and disrupts PAR1–Cav1 coassociation. However, the mechanisms that mediate PAR1–Cav1 interaction are not known, as PAR1 appears to lack caveolin binding motifs. Thus, we speculate that an intermediary protein may be critical for facilitating PAR1–Cav1 basal interaction and modulation after APC treatment. Nonetheless, these findings suggest that APC-induced Y14 phosphorylation of Cav1 alters its interaction with PAR1 molecules in caveolae (Fig. 9).

GRK5 is also critical for promoting APC-activated PAR1 phosphorylation and βarr2-mediated endothelial cytoprotective signaling (31). However, unlike PAR1, GRK5 harbors a previously described N-terminal caveolin binding motif with aromatic-rich residues that directly interacts with the Cav1 CSD *in vitro*, and Cav1 has been shown to inhibit GRK5 activity (27). The N-terminal caveolin binding motif is conserved across all GRKs (27). However, to our knowledge, the localization of endogenous GRK5 to caveolae and potential interaction with Cav1 in intact cells have not been reported. In this study, we found that endogenous GRK5 is present in Cav1-enriched fractions by sucrose density gradient fractionation. Moreover, ∼38% of the endogenous GRK5 population was directly visualized to colocalize with Cav1 using super-resolution STORM imaging. Thus, like PAR1, endogenous GRK5 localizes to caveolae in endothelial cells. In addition, APC activation of PAR1 appears to transiently disrupt colocalization of a small population of GRK5–Cav1 molecules, causing GRK5–Cav1 dissociation and reassociation within 30 min, as assessed by STORM imaging. However, we could not verify the effects of APC on the modulation of GRK5–Cav1 complexes using co-IP assays, although the trend of GRK5–Cav1 interaction is consistent with the STORM imaging studies. In addition, it is unclear whether the change in GRK5–Cav1 colocalization is related to Cav1 Y14 phosphorylation. Regardless, we hypothesize that disruption of the GRK5–Cav1 complex may permit APC-induced GRK5 activation to facilitate phosphorylation of PAR1, which is critical for βarr2 recruitment (31) and c-Src activation (Fig. 9). In a rodent model, Cav1 Y14 phosphorylation was shown to increase GRK2–Cav1 interaction and to decrease endothelial nitric oxide synthase activity in sinusoidal endothelial cells (46), consistent with Cav1 negative regulation of GRK activity. These findings raise the possibility that GRK5–Cav1 interaction may negatively regulate GRK5 activity in intact cells, but this remains to be experimentally tested.

GRK5 is primarily anchored at the plasma membrane *via* a C-terminal amphipathic helix (23, 24, 25). Unexpectedly, we found that the GRK5 IFF mutant, which lacks interaction with Cav1, is also defective in membrane association, whereas the GRK5 4A amphipathic helix mutant localizes primarily in the cytoplasm but retains Cav1 binding. These findings indicate that additional determinants of plasma membrane localization are perturbed in the GRK5 IFF mutant. In addition to the C-terminal amphipathic helix, the GRK5 N-terminal basic region binds to the plasma membrane lipid phosphatidylinositol 4,5-bisphosphate (47, 48) and may contribute to plasma membrane localization. Moreover, GRK5 dimerization mediated by an N-terminal short sequence may form a hydrophobic dimeric interface that may facilitate multivalent interaction with plasma membrane lipids, enhancing plasma membrane localization (49). Whether the GRK5 IFF mutant affects phosphatidylinositol 4,5-bisphosphate lipid binding or dimerization is not known. It is also possible that mutation of the GRK5 IFF motif perturbs the structural integrity of the RH domain and thereby perturbs its capacity to bind to the plasma membrane and to interact with caveolin-1.

The ubiquitously expressed β-arrestin-1 and -2 subtypes differentially regulate PAR1 signaling bias in response to thrombin and APC (31). Thrombin-activated PAR1-induced G protein signaling is dependent on βarr1 and not βarr2 for termination of signaling (50, 51), whereas APC-activated PAR1 signaling is driven by βarr2 and not βarr1 in endothelial cells (1). We showed that βarr2 expression is essential for propagation of APC-activated PAR1–induced Rac1 activation and not thrombin-induced Rho activation (1) and for APC-promoted Akt prosurvival signaling in endothelial cells (8). These findings suggest that βarr2 is essential for propagating APC-activated PAR1 signaling in endothelial cells despite the presence of βarr1 being detected in Cav1-enriched fractions (1). In addition, we recently reported that APC-induced βarr2 activation and conformational changes are distinct from thrombin (31). A critical function for βarr2 as the major transducer for APC cytoprotection in a stroke model has also been demonstrated *in vivo* (10). Together, these studies suggest that βarr2 functions as the main driver of APC-activated PAR1 cytoprotective signaling, whereas βarr1 does not appear to regulate PAR1 signaling or trafficking. Two studies previously demonstrated that APC-activated PAR1 fails to internalize from the cell surface in endothelial cells even after prolonged APC treatment (16, 52). Thus, the mechanism responsible for controlling APC-activated PAR1 signaling is not likely to be mediated by receptor trafficking but rather may result from modulating protein–protein interactions that could be mediated by Cav1 phosphorylation, which requires further interrogation.

In summary, this study shows that APC modulates Cav1 Y14 phosphorylation through a βarr2- and c-Src-dependent pathway and promotes dissociation of PAR1–Cav1 protein–protein interactions. We further demonstrate that APC alters a small population of colocalized GRK5–Cav1 molecules, but whether this is similarly regulated by Cav1 phosphorylation remains to be determined. In addition, GRK5–Cav1 interaction was shown to occur through an N-terminal Cav1 binding motif in intact cells. This GRK5 mutant is defective in Cav1 binding and plasma membrane localization and fails to support APC-activated PAR1–induced βarr2 recruitment. Collectively, our findings suggest that Cav1 function extends beyond its structural role and participates in protein–protein interactions with PAR1 and GRK5, key effectors of the APC-activated PAR1 cytoprotective signaling pathway (Fig. 9).

## Experimental procedures

### Cell culture

Endothelial EA.hy926 cells (#CRL-2922) were obtained from the American Type Culture Collection, authenticated by short tandem repeat profiling, and cultured as previously described (8). Briefly, cells were grown at 37 °C, 8% CO_2_ in Dulbecco's modified Eagle's medium (DMEM) from Gibco (#10-013-CV and #10437-028) supplemented with 10% fetal bovine serum (FBS) and 20% preconditioned media. HEK293T cells, HEK293A parental cells, GRK KO cells, GRK5,6 KO cells (from Dr Asuka Inoue, Tohoku University), and HEK293 Cav1,2 KO cells (from Dr Tracy Handel, UC San Diego) were cultured in DMEM supplemented with 10% FBS (v/v) with a 5% CO_2_ atmosphere at 37 °C. HeLa cells were cultured in DMEM supplemented with 10% FBS and 250 μg/ml hygromycin for maintenance as previously described (53).

### Antibodies

In this study, the following antibodies were used: mouse anti–PAR1 WEDE (Beckman Coulter, #IM2584), anti-Cav1 (CST, #3267S and BD, #610060), anti-Cav1 Y14 phospho antibody (CST, #3251), anti-βarr2 (Abcam, #ab54790), GAPDH (GeneTex, #GT239), c-Src Y416 (CST, #2101), anti-c-Src (CST, #2109), GRK5 (Santa Cruz, #sc-518005), GRK5 polyclonal antibody (Invitrogen, #PA5-96262) anti-GRK4-6 (Millipore, #05-466), anti-HA (CST, #3724S), anti-rabbit IgG (CST, #2729), anti-β-Tubulin (CST, #86298), anti–early endosome antigen-1 (BD Biosciences, #610457), anti-Vinculin (Sigma, #V9131), and anti-HA conjugated to horseradish peroxidase (Roche, #11667475001). The following secondary antibodies were used: anti-mouse or anti-rabbit horseradish peroxidase–conjugated antibodies (Bio-Rad, #170-6516 and #170-6515, respectively), anti-mouse Alexa-488 Fluor (Invitrogen, #A-11001), anti-rabbit Alexa-488 Fluor (Invitrogen, #A-11034), anti-rabbit Alexa-594 Fluor (Invitrogen, #A-11012), anti-mouse Alexa-594 Fluor (Invitrogen, #A-11032), anti-rabbit Alexa-647 Fluor (Invitrogen, #A-21244), and anti-mouse Alexa-647 Fluor (Invitrogen, #A-21235) antibodies.

### Agonists and inhibitors

The agonists used in these studies were human APC (Prolytic Haematologic Technologies LLC, #HCAPC-0080); human APC S360A proteolytically inactive mutant, kindly provided by Professor John Griffin (The Scripps Research Institute); human α-thrombin (Enzyme Research Laboratories, #HT1002a); and dasatinib (Sigma (#SML2589).

### Plasmids

Human FLAG-tagged PAR1 complementary DNA (cDNA) was previously described (54). GRK5 WT and 4A mutant in pcDNA3 were generously provided by Dr Philip Wedegaertner (Thomas Jefferson University, Philadelphia, PA). The GRK5 IFF mutant, PAR1-YFP, EPCR-Halo, and RLuc-βarr2 were generated by Gibson assembly homologous recombination (Gibson Assembly Master Mix; New England Biolabs), followed by whole plasmid sequencing. Cav1 containing a carboxyl-terminal HA-epitope tag (HA-Cav1) was a gift from Ari Helenius (Addgene plasmid #27703; http://n2t.net/addgene:27703; Research Resource Identifier: Addgene_27703) (55).

### siRNAs

The following siRNAs were used in the study, including c-Src siRNA Hs_SRC_11 5′-GGCGCGGCAAGGTGCCAAATT-3′, custom βarr2 siRNA #666 5′-GGACCGCAAAGTGTTTGTG-3′, and AllStars negative control nonspecific siRNA 5′-CUACGUCCAGGAGCGCACC-3′ and purchased from Qiagen. Cav1 siRNA 5′-CCCACTCTTTGAAGCTGTTGGGAAA-3′ was from Invitrogen, #CAV1HSS141467.

### Signaling assays and immunoblotting

Endothelial EA.hy926 cells were seeded at 3 × 10^5^ cells per well in a 12-well plate and serum starved overnight in DMEM containing 0.4% FBS. Cells were washed and incubated in serum-free DMEM supplemented with 1 mg/ml BSA, 10 mM Hepes, and 2.8 mM CaCl_2_ for 30 min, followed by a 30 min pretreatment with 50 nM dasatinib or dimethyl sulfoxide as a vehicle control and then stimulated with or without 20 nM APC for various times. Cell lysates were collected in 2X Laemmli sample buffer (LSB), supplemented with 200 mM DTT, and immunoblotted as indicated. EA.hy926 cells were seeded at 1.5 × 10^5^ cells per well in a 12-well plate, grown overnight, and transfected the next day with 50 nM siRNAs using the TransIT-X2. After 48 h, cells were starved overnight in DMEM containing 0.4% FBS. Cells were then washed and starved for 1 h with DMEM supplemented with 1 mg/ml BSA, 10 mM Hepes, and 2.8 mM CaCl_2_. Cells were then left untreated or treated with 20 nM APC, and cell lysates were collected in 2X LSB supplemented with 200 mM DTT. Equivalent amounts of cell lysates were then immunoblotted as indicated and quantified by densitometry using ImageJ software (National Institutes of Health). Quantification of protein phosphorylation was determined by normalizing to the corresponding total protein of the same sample and expressed as a fold over the indicated control group. Changes in expression of specific proteins in siRNA-transfected cells were normalized to a total protein loading control and expressed as a fraction of the indicated control group.

### Immunoprecipitation and immunoblotting

IP was carried out as described (8). Briefly, endothelial EA.hy926 cells were seeded at 4.95 × 10^6^ cells per 10-cm dish, grown overnight, and then serum starved for 1 h prior to treatment with 20 nM or 50 nM APC or 10 nM thrombin. In some experiments, cells were pretreated with 50 nM dasatinib or dimethyl sulfoxide as a vehicle control for 30 min prior to agonist stimulation. Cells were lysed with Triton lysis buffer (50 mM Tris [pH 7.4], 100 mM NaCl, 5 mM EDTA, 1% v/v Triton X-100, 50 mM NaF, and 10 mM NaPP) supplemented with protease inhibitors, clarified by centrifugation, and protein concentrations were determined by bicinchoninic acid (Thermo Fisher Scientific, #23221). Equivalent amounts of lysates were then precleared with Protein A-Sepharose (Sigma, #GE17-0780-01) and then incubated with Protein A beads and either anti-PAR1 WEDE antibody or anti-GRK5 antibodies (Santa Cruz, #sc-518005) and incubated at 4 °C overnight. IPs were collected, washed, and eluted in 2x LSB containing 200 mM DTT and resolved by SDS-PAGE, transferred to polyvinylidene fluoride membranes, immunoblotted, and developed by chemiluminescence and quantified by densitometry using ImageJ software. Changes in protein IP were determined by normalizing to the total protein of the same sample or loading control for siRNA-transfected cells and expressed as a fraction relative to the indicated control group.

HEK293 cells were seeded at 1 × 10^6^ cells per 6-cm dish and grown overnight and then transfected with 1 μg of either GRK5 WT, IFF, or 4A mutant or pcDNA3 cDNA plasmids along with 1 μg of HA-tagged Cav1 using polyethylenimine. Cells were lysed in Triton lysis buffer, and lysates were clarified by centrifugation for 20 min, quantified by bicinchoninic acid, and equivalent amounts of lysates were precleared and incubated with Protein A beads together with IgG or anti-HA antibody at 4 °C overnight. GRK5 IPs were quantified by densitometry using ImageJ software and normalized to the total corresponding GRK5 from the same sample and expressed as a fold over IgG control.

### Immunofluorescence confocal microscopy

HeLa cells were seeded on 12-mm coverslips precoated with 1 to 5 μg/cm^2^ fibronectin (Sigma, F1141) in 24-well plates at a density of 0.2 × 10^6^ cells per well and grown overnight at 37 °C. Cells were then transfected with either 1 μg of GRK5 WT, IFF, or 4A mutants or pcDNA3.1 cDNA plasmids. After 24 h, cells were washed with ice-cold PBS, fixed with 4% paraformaldehyde for 5 min, permeabilized with cold MeOH for 30 s, and immunostained with anti-GRK5 antibody (Santa Cruz, #sc-518005) at 1:100 dilution and followed by anti-mouse Alexa-488 antibody. Coverslips were mounted with ProLong Gold Antifade Mountant (Invitrogen, #P10144). Confocal images of 0.20-μm x–y sections were acquired sequentially using an Olympus IX81 DSU spinning-disk microscope equipped with a 60x Plan Apo objective lens (numerical aperture = 1.4), along with appropriate excitation–emission filters and a Cool SNAP HQ2 CCD camera (Andor) using Metamorph, version 7.7.4.0 software (Molecular Devices). Image line scan analysis was performed using Fiji ImageJ.

To validate antibodies for STORM imaging, HEK293 cells were seeded on coverslips in 24-well plates at a density of 0.1 × 10^6^ cells per well and transfected with 1 μg of PAR1 in pcDNA3 or pcDNA3 only. Cells were fixed as described above and then immunostained with anti-PAR1 WEDE antibody, followed by secondary anti-mouse Alexa-488 antibody. The Cav1 and GRK5 antibodies were validated using HEK293 parental cells and CRISPR–Cas9 GRK KO, GRK5,6 KO, or Cav1,2 KO cells grown on coverslips in 24-well plates at a density of 0.2 × 10^6^ cells per well and incubated with the Cav1 antibody (CST, #3267S) or GRK5 antibody (Santa Cruz, #sc-518005), followed by secondary anti-rabbit Alexa-488 antibody or anti-mouse Alexa-488 antibodies, mounted and imaged by confocal microscopy as described above.

### STORM–TIRF imaging and analysis

STORM imaging was used to analyze colocalization between endogenous PAR1–Cav1 and GRK5–Cav1 molecules in endothelial cells using the validated antibodies. Cells plated in 35-mm glass-bottom dishes were grown in DMEM containing 0.4% FBS overnight and serum starved in DMEM containing 1% BSA and 20 mM Hepes for 1 h at 37 °C. Cells were then treated with or without 20 nM APC as indicated. Cells were fixed with 4% paraformaldehyde, permeabilized, and incubated with either anti-PAR1 or anti-GRK5 antibodies together with anti-Cav1. Cells were then incubated with either mouse or rabbit Alexa Fluor-488 antibodies, followed by anti-Alexa Fluor 647 secondary antibodies to detect Cav1, respectively. Cells were then immersed in STORM imaging buffer (50 mM Tris, pH 8.0, 10 mM NaCl, 10% glucose, 0.1 M cysteamine [#30070-50G, Sigma–Aldrich]), 0.56 mg/ml glucose oxidase (#G2133-250KU, Sigma–Aldrich), and 0.68 mg/ml catalase (#C40-100MG, Sigma–Aldrich). STORM imaging was conducted using a Nikon A1R Confocal STORM microscope system equipped with a 100X/1.49 numerical aperture TIRF oil-immersion objective and an ANDOR iXon3 Ultra DU897 EMCCD camera (UC San Diego Moores Cancer Center). Data acquisition was performed using Nikon NIS-Elements AR v5.21.01 software in multicolor continuous mode. Single-molecule localization was achieved by rapid stochastic sequential laser (488 and 647 nm) activation and deactivation of a small subset of fluorophores at optimized power settings to enable localization of individual molecules. Sample drift was corrected using the software's autocorrelation-based drift correction algorithm, and axial drift was minimized with the Nikon Perfect Focus System. Image acquisition was typically 1 to 2 million cycles of single-molecule imaging and detection. Reconstruction of super-resolution images, molecule localization, visualization, and data analysis were conducted using the NIS-Elements N-STORM Analysis module. Sample preparation for STORM image acquisition was conducted according to the N-STORM Protocol-Sample Preparation guide and Dempsey *et al.* (56).

### Sucrose density gradient fractionation

Sucrose fractionation was carried out as previously described (8). EA.hy926 cells seeded at 4.95 × 10^6^ cells per 10-cm dish were grown overnight, rinsed with ice-cold PBS, and lysed in a sodium carbonate buffer (150 mM sodium carbonate at pH 11, 1 mM EDTA, and protease inhibitors). The lysates were processed by 10 strokes with a Dounce homogenizer, passed through an 18-gauge needle, and sonicated on ice (Branson Ultrasonics Corp). Cell lysates (800 μl) were mixed with an equal volume of 80% sucrose in MES-buffered saline supplemented with 300 mM sodium carbonate and placed in a 12-ml ultracentrifuge tube. Density gradients were created by adding 6 ml of 35% MES-buffered saline with 150 mM sodium carbonate on top and 4 ml of 5% sucrose in MES-buffered saline with 150 mM sodium carbonate on top of that. The samples were ultracentrifuged in an SW41 rotor for 20 h at 4 °C with a force of 23,000*g*, and 1 ml fractions were collected sequentially, resolved by SDS-PAGE, and immunoblotted as indicated and quantified by densitometry using ImageJ software.

### Cell fractionation assay

HEK293 cells were transfected with GRK5 WT, IFF, 4A, or pcDNA3 plasmids and grown on 60 mm dishes overnight. Cells were collected and suspended in detergent-free hypotonic buffer (50 mM Tris–HCl, pH 8, 2.5 mM MgCl_2_, and 1 mM EDTA) supplemented with protease inhibitors. Cells were sheared using a syringe with a 21-gauge needle 10 times, and cell lysates were centrifuged at 400*g* for 5 min. Supernatants were collected and subjected to ultracentrifugation at 150,000*g* for 20 min to isolate soluble/cytosolic fractions from particulate/membrane fractions as described (49). Equivalent amounts of soluble and particulate fractions were analyzed by immunoblotting and quantified by densitometry using ImageJ software.

### BRET assay

HEK293 GRK KO cells were seeded at 5.0 × 10^5^ cells per well in a 6-well plate, grown overnight, and transfected with PAR1-YFP (1000 ng), EPCR (500 ng), Rluc–βarr2 (250 ng), and either GRK5 WT, IFF, or 4A mutant, or pcDNA3.1 (125 ng). The following day, cells were reseeded into 96-well plates coated with poly-d-lysine at a concentration of 3 × 10^4^ cells per well and grown overnight. The cells were then washed with PBS and serum starved for 1 h using a 1:1 mixture of DMEM without phenol red and PBS. After the starvation, cells were preincubated with the RLuc substrate Coelenterazine H (5 μM) for 5 min and then stimulated with 20 nM APC. BRET measurements were taken using a Berthold TriStar LB941 multimode plate reader equipped with Micro WIN 2010 software using two filter settings: 480 nm for Rluc and 530 nm for YFP. The BRET signal was calculated as the emission at 530 nm divided by the emission at 480 nm. The BRET signals were then normalized to basal BRET ratios and expressed as a percentage over the basal level. The data were fit against a one-phase association nonlinear regression model, and the area under the curve was calculated using GraphPad Prism 10.4.1 (GraphPad Prism software).

### Statistical analysis and software

Data were analyzed using Prism 10.4.1 (GraphPad Software) and Microsoft Excel software. Statistical analysis was conducted using Student's *t* test, one-way or two-way ANOVA, followed by post hoc tests for data (mean ± SD) of three or more independent biological replicates, as indicated in the figure legends. Figures were created in Adobe Illustrator, and cartoons were created with BioRender.com.

## Data availability

All data are contained within the article.

## Conflict of interest

The authors declare that they have no conflicts of interest with the contents of this article.
